# Annot: a Django-based sample, reagent, and experiment metadata tracking system

**DOI:** 10.1186/s12859-019-3147-0

**Published:** 2019-11-01

**Authors:** Elmar Bucher, Cheryl J. Claunch, Derrick Hee, Rebecca L. Smith, Kaylyn Devlin, Wallace Thompson, James E. Korkola, Laura M. Heiser

**Affiliations:** Department of Biomedical Engineering and OHSU Center for Spatial Systems Biomedicine, OHSU, Portland, OR 97201 USA

**Keywords:** Controlled vocabulary, Annotation, Metadata, Software, Annotamentum

## Abstract

**Background:**

In biological experiments, comprehensive experimental metadata tracking – which comprises experiment, reagent, and protocol annotation with controlled vocabulary from established ontologies – remains a challenge, especially when the experiment involves multiple laboratory scientists who execute different steps of the protocol. Here we describe Annot, a novel web application designed to provide a flexible solution for this task.

**Results:**

Annot enforces the use of controlled vocabulary for sample and reagent annotation while enabling robust investigation, study, and protocol tracking. The cornerstone of Annot’s implementation is a json syntax-compatible file format, which can capture detailed metadata for all aspects of complex biological experiments. Data stored in this json file format can easily be ported into spreadsheet or data frame files that can be loaded into R (https://www.r-project.org/) or Pandas, Python’s data analysis library (https://pandas.pydata.org/). Annot is implemented in Python3 and utilizes the Django web framework, Postgresql, Nginx, and Debian. It is deployed via Docker and supports all major browsers.

**Conclusions:**

Annot offers a robust solution to annotate samples, reagents, and experimental protocols for established assays where multiple laboratory scientists are involved. Further, it provides a framework to store and retrieve metadata for data analysis and integration, and therefore ensures that data generated in different experiments can be integrated and jointly analyzed. This type of solution to metadata tracking can enhance the utility of large-scale datasets, which we demonstrate here with a large-scale microenvironment microarray study.

## Background

A typical biological experiment entails perturbation of a model biological system (e.g.*,* cell line) with a treatment of interest (e.g., drug or growth factor) followed by assessment of molecular or phenotypic changes. A critical aspect of such experiments is the collection of key metadata required to interpret and analyze the resultant data. Such detailed information about samples, reagents, and protocols is challenging to collect for complex experiments, particularly when they involve multiple laboratory scientists who execute different steps. Recently, the scientific community has recognized the need for detailed metadata reporting as a cornerstone of reproducible experiments [1, 2]. This is further motivated by the explosion of large-scale datasets that can be used in integrative analysis only if they are associated with complete and accurate metadata that adequately describe the experiment [3–8].

Several efforts have been made to aid reproducibility, including: ontology-based controlled vocabulary [9, 10], minimal information guidelines [11], standardized metadata annotation formats [12], and creation of programming language libraries to standardize and automate protocols [13]. Despite these resources, robust, facile, and comprehensive metadata tracking continues to be a challenge in the biological sciences, and there remains a need for software that allows metadata collection using controlled vocabulary and structured formats appropriate for downstream analyses. Here we describe Annot, a novel web application to track highly structured sample, reagent, and assay metadata. Annot was designed to be adaptable to diverse experimental assays and accordingly has broad applicability to the research community.

### Implementation

Our overarching goal was to create a database to support the collection and access of controlled, structured experimental metadata to meet the needs of both computational and experimental scientists. The development of Annot was motivated by the need to annotate reagents and samples in compliance with LINCS data standards [2], including annotation of spotted arrays, and tracking reagent and cell lines to the lot and passage number level. We chose to develop a web framework so that the database would be easily accessible to staff throughout the laboratory. Moreover, this provides a path to implement additional functionality for various tasks, including: loading standard ontologies, exporting metadata files, and system backup.

The final version of Annot implemented the web framework with Django and leveraged its associated libraries. Django’s admin library provides a robust GUI for the database and the Django-selectables library was used to create searchable drop-down menus. Django web framework provides basic security measures. For example, access to view, add, or change entries can be restricted for each database table and user. Django also protects against common attacks such as SQL injections, cross-site scripting, cross-site request forgery, and clickjacking. Finally, data quality can be monitored by inclusion of a field that indicates the user who entered the information. We used Postgresql as the database backend, which was connected to the web framework by the Psycopg2 library; interaction with the database occurs via Django’s object-relational mapper (ORM). The web server is Nginx, which was connected to the web framework by the Gunicorn library.

We ensured that Annot would be easy to maintain and deploy through the use of the Docker platform. Specifically, the Annot code base (Fig. 2), Postgresql database, Nginx webserver, and data storage file system can be run in separate Docker containers that are orchestrated via Docker-compose. The whole Docker engine is spun up with Docker-machine and utilizes Virtualbox as a Docker-machine disk drive. With dockerization, version updates to each part of the system are facilitated by re-building and deploying the particular container.

Annot utilizes several controlled vocabulary ontologies that cover diverse aspects of biological experiments (Table 1). We employed the Requests library to automate updating of controlled vocabulary with the current ontology version. For each controlled vocabulary term, Annot automatically stores the most recent ontology version, which can be retrieved through the user interface.

**Table 1:** controlled vocabulary usage and source overview

Ontology	Vocabulary	Source	Official Url
BAO	clonality, genetic modification, growth property, immunology isotype, transient modification	bio ontology	http://www.bioassayontology.org/
DOID	disease	bio ontology	http://diseaseontology.sourceforge.net/
EFO	unit	bio ontology	http://www.ebi.ac.uk/efo
MESH	cell type	bio ontology	http://www.nlm.nih.gov/mesh/meshhome.html
NCBITAXON	organism	bio ontology	http://www.ncbi.nlm.nih.gov/taxonomy
OBI	sex	bio ontology	http://purl.obolibrary.org/obo/obi
SNOMEDCT	antibody part, ethnicity	bio ontology	http://www.ihtsdo.org/
UBERON	organ, tissue	bio ontology	http://uberon.org/
CELLOSAURUS	sample	cellosaurus	http://web.expasy.org/cellosaurus/
CHEBI	compound	ebi	http://www.ebi.ac.uk/chebi
ENSG	gene	ensembl	http://www.ensembl.org/index.html
GENEONTOLOGY	gene ontology, biological process, cellular component (protein complex), molecular function	gene ontology	http://geneontology.org/
UNIPROT	protein	uniprot	http://www.uniprot.org/proteomes/
OWN	dye, health status, provider, sample entity, verification profile, yield fraction	own	https://gitlab.com/biotransistor/annotTutorial/

## Results

**Fig. 1:**
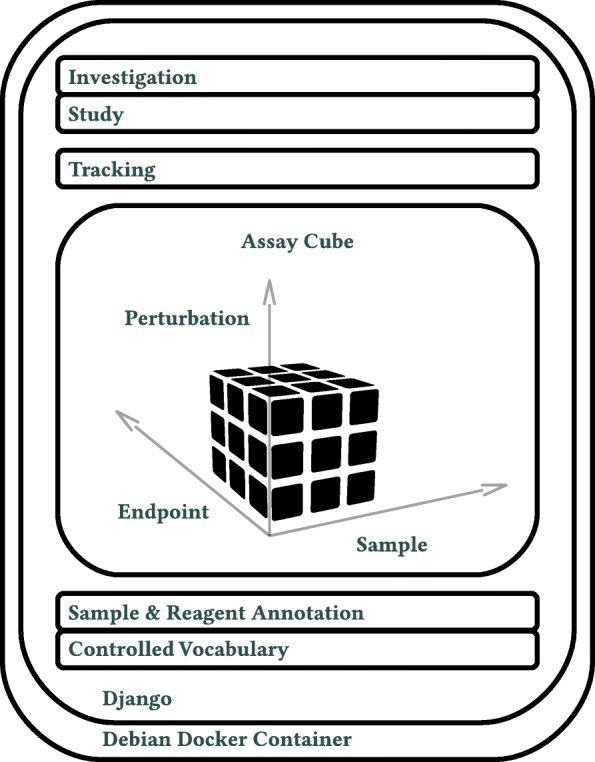
Schematic representation of the Annot Django code base stack. Annot was designed in a bottom-up approach. The controlled vocabulary layer establishes the basis for sample and reagent annotation in the brick layer. In the assay cube layer, sample and reagent bricks can be assembled into any experimental layout using Python3 scripting. In the tracking layer, assays can be annotated with protocols, execution dates, and staff. In the uppermost layers, studies and investigations can be used to group assays. Each layer depends on lower layers but not on upper layers

**Fig. 2:**
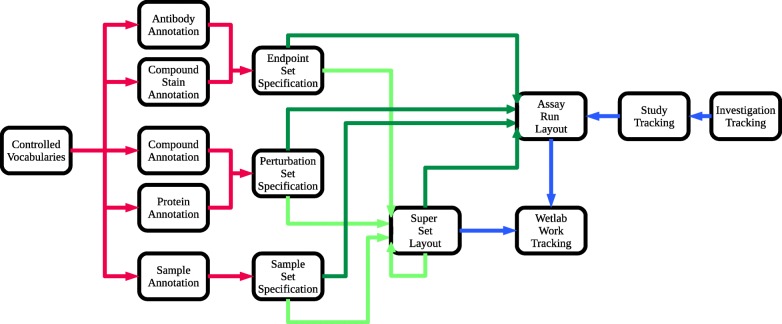
Annot workflow representation. Assay reagents and samples are first annotated via the Annot web interface or via Excel spreadsheet that can be uploaded into Annot. This annotation step enforces the use of controlled vocabulary and official gene, protein, compound and cell line identifiers. Annotated reagents and samples are next combined into endpoint, perturbation, and sample sets. In this step, additional experimental details can be specified, for example, reagent concentrations, cell seeding density, or cell passage number (red arrows). For assays that involve robot pipetting, array spotting, or cyclic staining, super sets can be generated (light green arrows). Finally, for each assay: run, endpoint, perturbation, sample, and super sets are merged to a run specific assay layout (dark green arrows). Assays and supersets that are regularly processed by the lab can be directly tracked in Annot, along with specification of the date, protocol and laboratory personnel. Lastly, assays can be grouped into studies and studies into investigations (blue arrows)

We implemented Annot as a layered hierarchy to capture essential metadata elements used in biomedical experiments (Figs. 1, 2). The base layer contains controlled vocabulary – defined by established ontologies – for sample and reagent annotations. Terms not represented in existing ontologies can be added and archived such that they will not interfere with controlled ontology terms. Controlled vocabulary from the base layer is used to annotate sample and reagent “annotation bricks” that specify the metadata for each entry; these entries are held in a database table. The current version of Annot includes annotation brick tables for human cell lines, compounds, proteins, protein complexes, antibodies, and cell stains. To ensure clarity, Annot fuses the corresponding ontology identifier (e.g. UniProt protein identifier) to a human readable identifier (e.g., HUGO gene symbol).

In the middle layer, curated annotation bricks are linked together to describe each experimental condition and the experimental design. The cornerstone of this middle layer is an adaptation of the input-processing-output (IPO) model from software engineering, which is used to describe the structure of an information processing program. In the biological experiment use-case, these key features are sample (input), perturbation (processing), and assay endpoints (output), which can be represented as axes on a cube (Fig. 1). Each of these axes is annotated to create a complete record of the experimental protocol. These metadata are stored in a json syntax-compatible format, termed acjson, which our self-contained Python3 acpipe_acjson library (https://gitlab.com/biotransistor/acpipe_acjson) can produce and process. Next, the tracking layer enables assay-related date, protocol, and staff member metadata to be documented. Finally, in the top layers, assays can be grouped into studies and studies grouped into investigations. This permits distinct assays to be linked together. Altogether, this approach ensures robust metadata that can be used in downstream analyses.

Annot’s modular implementation provides flexibility and ease-of-use. For example, all layers can be downloaded as tab-delimited and json files, with the latter format used in backups. Metadata from assays can be downloaded as acjson files, layout files (i.e., spreadsheets), or dataframe files that can be easily ported to Pandas or R for analysis. Additionally, annotation bricks created in tab-delimited format as spreadsheets can be directly loaded into the annotation brick tables; these are checked against controlled vocabulary terms to ensure database integrity. Finally, the tracking layer can be adapted to a laboratory’s specific needs, for example: to add or swap out ontologies, or to implement new annotation brick tables to capture experiment-specific metadata. To support the user community, we created several resources, including a Gitlab page with bug reporting and an online user manual: https://annot.readthedocs.io/en/latest/. This documentation includes a detailed tutorial based on data from an experiment performed in our laboratory, a project discussion section, a HowTo section, and a Software Reference section.

As proof-of-concept, we used Annot to track metadata for a large-scale microenvironment microarray (MEMA) experiment [14]. This complex experimental protocol involves fabrication of microarrays spotted with insoluble extracellular matrix proteins, cell culturing, and treatment of cells with growth factors and therapeutic compounds, followed by fixing, staining, and imaging cells on a fluorescent microscope [15]. All aspects of the experiment were recorded in Annot, including detailed cell line and reagent metadata, experimental protocols, and execution dates. The data and metadata are publically available for download (https://www.synapse.org/MEP_LINCS) and are used as an example case in the Annot tutorial (https://annot.readthedocs.io/en/latest/man_tutorial.html). Using Annot, we were able to track all aspects of the experiment using controlled vocabulary in compliance with LINCS standards [2].

## Discussion

Our major goal was to develop a software platform to track the essential metadata of an assay so that data generated across different experiments can be integrated and jointly analyzed. The common solution to this in biological research laboratories is to employ spreadsheets. While spreadsheets benefit from being flexible and easily edited, they are subject to errors that result from manual entry, inadvertent auto-formatting [16], and version drift. Importantly, Annot offers a robust solution to annotate – using controlled vocabulary – samples, reagents, and experimental details for established assays where multiple staff are involved. A major strength of Annot is that it is designed to manage metadata for complex experimental paradigms where the use of spreadsheets would be too tedious and error-prone. It is particularly useful for laboratories that do large-scale data production and run the same assay repeatedly, as it permits multiple scientists to annotate the various aspects of the assay to which they contributed.

Annot can store detailed information about diverse reagents and sample types, each defined by a “brick.” Key fields for bricks were informed by the LINCS data standard; however, Annot’s flexible design allows these bricks to be modified to suit a laboratory’s particular needs or for additional bricks to be added as required to fully annotate an experiment. Additionally, by specifying metadata for both reagents and samples, Annot enforces controlled vocabulary identifiers for genes, proteins, protein complexes, compounds, and cell lines, which is necessary for integration across assays. We are not aware of any other metadata annotation tool that enforces controlled annotation of all these classes of identifiers.

Several related annotation tools are available in the community, though Annot is unique in its ability to manage controlled metadata for complex experimental assays. ISAcreator [17] is similar to Annot in that it offers functionality for tracking investigations, studies, and assays in great detail; however, it was created for relatively simple experimental designs and cannot manage metadata for complicated assays such as MEMA [14, 15]. Other annotation tools are designed to manage specific metadata, such as the OME data model and file formats for biological images [18]. Laboratory management platforms such as the proprietary web service Quartzy (https://www.quartzy.com) can provide basic reagent annotation; however, such services typically do not enforce controlled vocabulary and identifiers. Protocols.io is a proprietary platform for sharing detailed wet lab protocols, which is another key aspect to transparency and reproducibility (https://www.protocols.io). Related to metadata management is sharing and annotation of data and analyses, and several hosted web applications are available for such purposes. For example, Synapse [19] from Sage Bionetworks and Open Science Framework (OSF, https://osf.io/) from the Center for Open Science are technology platforms designed to facilitate collaborative development of reproducible analytical pipelines. These tools serve purposes that are distinct from Annot, and we envision that they could be leveraged together with Annot to create a complete experimental and analytical workflow that complies with FAIR standards [1].

While Annot was written with an informatics agnostic end-user in mind, full system administration requires basic skills in Linux, Python3, and Django, as well as basic knowledge of relational databases. Because of the cost required to populate Annot with detailed sample and reagent annotation, it is most appropriate for large-scale, high-throughput experiments. However, a major benefit to our approach is that data generated in different experimental settings can be integrated through a detailed description of each experimental condition along the dimensions of sample, perturbation, and endpoint. Moreover, we surmise that the high cost of large-scale screening efforts warrants the time and effort required to ensure adequate annotation. Ultimately, approaches such as this will allow data to be better leveraged and utilized to make discoveries and biological insights.

## Conclusions

Detailed annotation of experimental metadata is an essential component of biomedical data, and is necessary to ensure accurate analysis and interpretation of such data. Annot offers a robust, flexible solution to annotate samples, reagents, and experimental protocols where multiple laboratory scientists are involved in running established assays. Annot can be adapted to different experimental paradigms and loaded with any relevant ontology, thereby ensuring use of controlled metadata. This framework can also be used to store and retrieve metadata for analysis and integration and therefore can enhance the utility of large-scale datasets.

## Availability and requirements

*Project name:* Annotamentum

*Project home page for source code:* https://gitlab.com/biotransistor/annot

*Project home page for user manual:* https://annot.readthedocs.io/en/latest/

*Operating systems:* Linux, MacOS, Windows

*Programming language:* Python3

*Other requirements:* Docker, Internet access, Web browser

*License:* GNU GPLv3

*Any restrictions to use by non-academics:* GNU GPLv3

## Data Availability

Tutorial and tutorial material to demonstrate the usability of the implementation are available at https://annot.readthedocs.io/en/latest/index.html and https://gitlab.com/biotransistor/annotTutorial.

## Abbreviations

FAIR: Findable accessible interoperable reusable
IPO: Input-processing-output
LINCS: Library of Integrated Network-based Cellular Signatures
MEMA: Microenvironment microarray
OME: Open microscopy environment
ORM: Object-relational mapper
OSF: Open science framework
